# A Novel Differential Time-of-Arrival Estimation Technique for Impact Localization on Carbon Fiber Laminate Sheets

**DOI:** 10.3390/s17102270

**Published:** 2017-10-03

**Authors:** Eugenio Marino Merlo, Andrea Bulletti, Pietro Giannelli, Marco Calzolai, Lorenzo Capineri

**Affiliations:** Department of Information Engineering, University of Florence, Via S. Marta 3, 50139 Firenze, Italy; eugenio.marinomerlo@unifi.it (E.M.M.); andrea.bulletti@unifi.it (A.B.); pietro.giannelli@unifi.it (P.G.); marco.calzolai@unifi.it (M.C.)

**Keywords:** piezoelectric ceramic sensors, structural health monitoring, piezopolymer sensors, CFRP laminates, impact localization

## Abstract

Composite material structures are commonly used in many industrial sectors (aerospace, automotive, transportation), and can operate in harsh environments where impacts with other parts or debris may cause critical safety and functionality issues. This work presents a method for improving the accuracy of impact position determination using acoustic source triangulation schemes based on the data collected by piezoelectric sensors attached to the structure. A novel approach is used to estimate the Differential Time-of-Arrival (DToA) between the impact response signals collected by a triplet of sensors, overcoming the limitations of classical methods that rely on amplitude thresholds calibrated for a specific sensor type. An experimental evaluation of the proposed technique was performed with specially made circular piezopolymer (PVDF) sensors designed for Structural Health Monitoring (SHM) applications, and compared with commercial piezoelectric SHM sensors of similar dimensions. Test impacts at low energies from 35 mJ to 600 mJ were generated in a laboratory by free-falling metal spheres on a 500 mm × 500 mm × 1.25 mm quasi-isotropic Carbon Fiber Reinforced Polymer (CFRP) laminate plate. From the analysis of many impact signals, the resulting localization error was improved for all types of sensors and, in particular, for the circular PVDF sensor an average error of 20.3 mm and a standard deviation of 8.9 mm was obtained.

## 1. Introduction

Interest in the detection and measurement of impact position on critical structures and components is growing in the scientific community. Composite materials are now commonly used in the aerospace, automotive, and transportation industries. Several authors have proposed impact localization methods based on acoustic/ultrasonic sensing. Kundu et al. have studied the minimization of transducers to be installed on the structure by leveraging the direction of arrival of the localized acoustic source [1]. Other authors have approached the problem using techniques based on the Continuous Wavelet Transform (CWT) [2,3,4,5].

The influence of medium anisotropy, which results in different propagation velocities along different directions, was investigated in [6]. Moreover, the dispersive behavior of guided (Lamb) waves can become a non-negligible phenomenon when dealing with impact response signals propagated over significant distances.

Structural Health Monitoring (SHM) provides real-time integrity monitoring of in-service structures to prolong their life and reduce maintenance costs. The Structure Health Monitoring system includes active sensing and passive sensing techniques: (1) passive sensing is where all of the sensors listen to the structure response caused by impacts to the structure. This becomes useful for analyzing impact events and impact force-time history; (2) active sensing is an excitation signal is sent to an actuator and other sensors pick up the structure response from the excitation signal. This type of sensing can detect damages on metal or composite structures including cracks, corrosion, delamination, and debonding, etc. By combining the active sensing and passive sensing technology together, a comprehensive scheme can be provided to detect any abnormally of the structure in real-time. Localization accuracy is important for correlating specific impact events with damage detection data and monitoring their progression over time, especially when using prognostic models [7].

In [8,9,10,11], the authors presented a guided-wave ultrasound structural health system based on linear arrays of interdigital piezopolymer transducers bonded to a composite pressure vessel. In that system, interdigital transducers were adopted to perform both damage assessment and impact detection/localization using a simple fixed-threshold technique [8,12]. 

The work hereby presented aimed at improving the impact localization performance of previous systems with novel signal analysis techniques.

In its most basic form, impact localization through triangulation is performed by observing the instants at which an impact response signal is recorded by (at least) three sensing elements placed at known positions, and inferring the source location by knowing the propagation velocity of each signal. Since the position of the sensing elements is generally fixed by design, the main sources of error in this calculation are the identification of the Differential Time-of-Arrival (DToA) between the sensors, and the propagation velocity of the impact response signal. The issues related to Lamb wave propagation velocity were already addressed in [8], however that work lacked a thorough investigation of the problem of reliably extracting the DToA from impact response signals affected by spurious contents.

When performing impact localization on a plate-like structure by detecting guided (Lamb) wave ultrasound signals, the problem is further complicated by a series of factors that are related to both the mechanical properties of the medium, and to the intrinsic characteristics of the propagating modes. The aim of this work was developing a versatile impact localization algorithm that could address both issues, and provide accurate and predictable results regardless of the sensor technology and with limited knowledge of the propagating modes group velocities into the material.

The research started by empirically investigating the behavior of a Carbon Fiber Reinforced Polymer (CFRP) laminate plate when subject to impacts. This helped in understanding the characteristics of impact response signals that were relevant to the process of impact localization.

Afterwards, the propagation velocity profiles of Lamb waves on the plate were characterized to understand the extent of their influence on impact response signals, and to extrapolate the data needed to perform a triangulation on such a structure. 

This preliminary experimental phase led to the development of a new method for extracting the DToA from the impact response signal, based on the identification and selection of specific features from these signals. The proposed DToA extraction method was thus integrated with the triangulation algorithm and evaluated with a series of controlled impact localization tests using different sensor technologies and impact energies.

The proposed approach is to generate a data base of impact signals for the following analysis of the average error and the standard deviation of each sensor type. We believe that such performance analysis based on an experimental database is not common in related works in the literature, and is very useful for the laboratory investigation of real composite material structures. 

The paper starts by describing the experimental setup used for performing impact tests and acquiring the response signals from the sensors. Impact response signals are analyzed in Section 3 to highlight those characteristics that allow the definition of the proposed DToA estimation approach. The characterization of guided-wave propagation on the plates is then presented in Section 4, and the improved impact localization algorithm is then presented is Section 5. Lastly, a set of validation experiments performed at various positions on the test plate, and recorded with different triplets of sensors, is reported, and the results are commented.

## 2. Experimental Setup

Repeatable impacts were generated on the test plate by a free-falling (unguided) metal sphere released by an electromagnet. The mass *m* of the impactor and the height *h* from the plate surface defined the impact energy according to the equation

(1) E=mgh

where *m* is the mass of the sphere, *g* is the gravitational acceleration and *h* the drop height. Considering *m* = 8.5 g, *g* = 9.8 m/s^2^ and *h* = 420 mm, the impact energy was *E* = 35 mJ. The precision of the impact test rig was evaluated using carbon paper to trace the actual point of impact of the falling sphere, resulting in a repeatability better than 4 mm.

**Figure 1:**
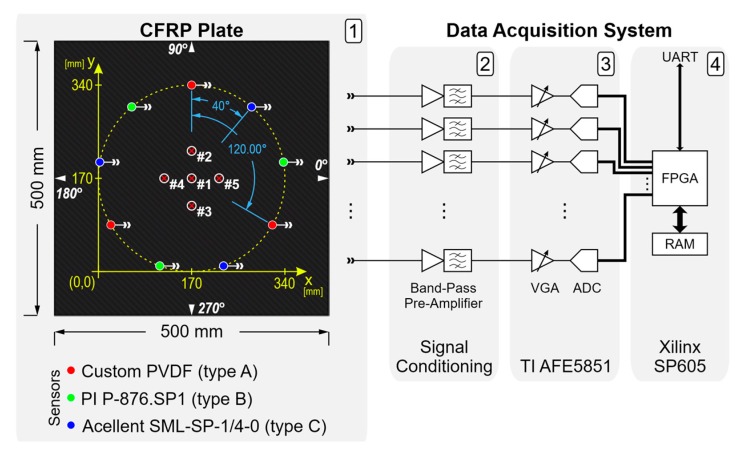
Block diagram of the data acquisition system and drawing of the test object: (**1**) Sensor layout on the Carbon Fiber Reinforced Polymer (CFRP) plate, coordinate reference system, and impact markers (#1, #2, #3, #4, #5), labels 0°, 90°, 180°, 270° indicate the angular directions used for measuring the Lamb wave group velocities; (**2**) signal conditioning electronics; (**3**) multichannel VGA with ADC evaluation module (Texas Instruments AFE5851EVM, Dallas, TX, USA); (**4**) Spartan-6 FPGA evaluation card (Xilinx SP605).

**Figure 2:**
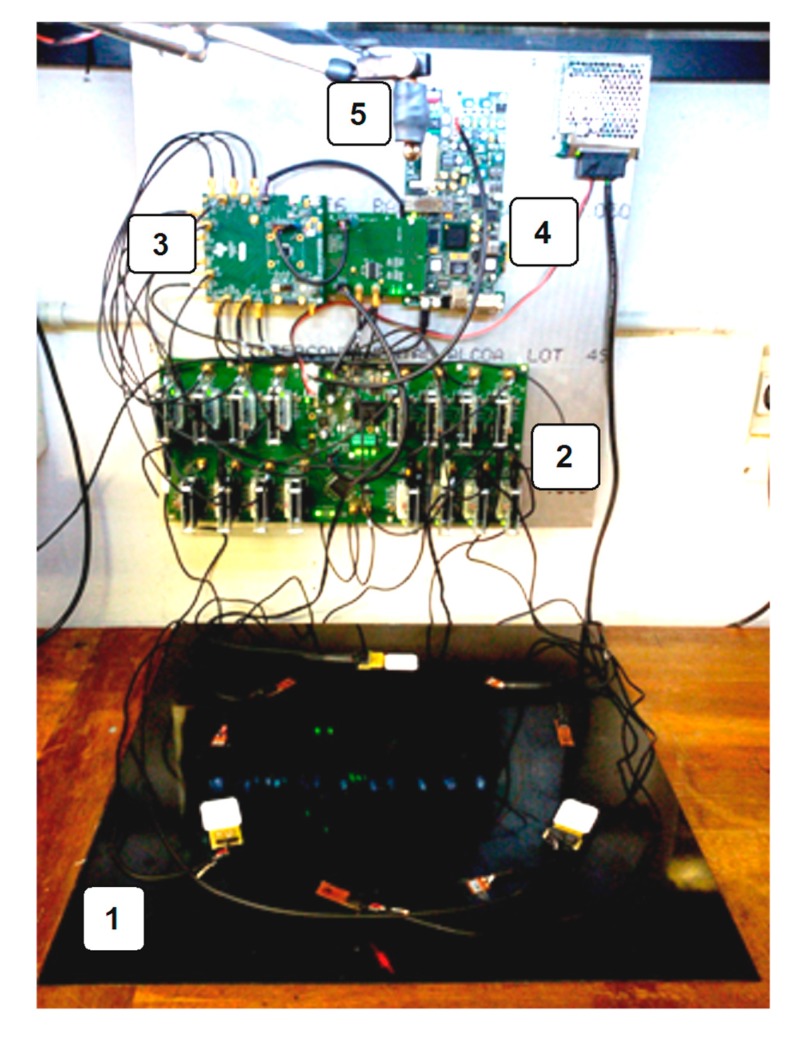
Picture of the experimental test bench: (**1**) CFRP test plate with all the sensors attached; (**2**) custom signal conditioning electronics; (**3**) multichannel variable gain amplifier (VGA) with ADC evaluation module (Texas Instruments AFE5851EVM); (**4**) Spartan-6 FPGA evaluation card (Xilinx SP605); (**5**) electromagnet retaining the impactor sphere.

The data acquisition system, described in [8], is shown in Figure 1 (block scheme) and Figure 2 (picture of the experimental setup). The front-end electronics provided an independent variable gain amplifier (VGA) per sensor channel.

The sampling rate was fixed at f_S_ = 10 MSps, with 10,000 samples acquired per trace (i.e., a 1 ms record length). A hardware impact detection procedure ensured that the time traces from all the channels were captured synchronously within a time window including at least 100 μs of signal before the triggering event occurred, which corresponded to the first leading wave detected by any one of the sensors (generally the one closest to the actual impact point). The data were then transferred to a computer and processed using MATLAB.

While the hardware impact detection procedure used a fixed threshold (in the order of dozen of mV) to capture the impact response within a certain time window, this information was later ignored by the proposed localization algorithm, and, as will be explained below, a different approach was used to extract a better estimate of the DToA from the recorded traces.

**Figure 3:**
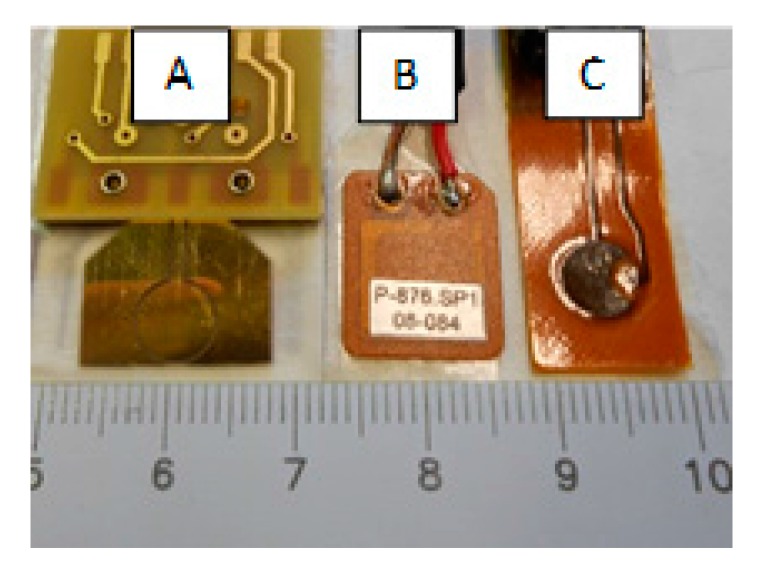
Picture of the three types of piezoelectric sensors used in this work. From left to right: custom circular PVDF (type A); Physik Instrumente P-876.SP1 (type B); and, Acellent SML-SP-1/4-0 (type C).

Impact tests were performed with three types of sensors triplets attached to the plate surface using double-sided tape (Tesa 4972) and interleaved at regularly spaced angles over a circle of diameter 340 mm, resulting in 120° spacing between sensors of the same kind (as shown in Figure 1). The three types of sensors, pictured in Figure 3, were: 3× custom-designed piezopolymer (PVDF) sensors (Type A) [13], 3× piezoceramic Physik Instrumente P-876.SP1 (Type B), and 3× piezoceramic Acellent SML-SP-1/4-0 (Type C). Each triplet of sensors was used independently of the others for impact localization. All of the selected sensors shared two important characteristics that made them suitable for these experiments: a flat response in the low-frequency range (no resonances below ~150 kHz), and similar dimension of the piezo-active area (~1/4).

The coordinate system depicted in Figure 1, which will be used throughout this paper to determine any position on the CFRP plates, has its origin defined by the lower left corner of the square circumscribed about the circle of sensors (and not an actual corner of the plate).

## 3. Analysis of Impact Response Signals on CFRP Plates

The CFRP plate used during the experiments had dimensions 500 mm × 500 mm and thickness 1.25 mm. It was a symmetric angle-ply laminate with a total of 10 plies having the following fiber orientation stack-up: 45°/0/−45°/45°/90°/90°/45°/−45°/0/45°. This construction resulted in quasi-isotropic mechanical characteristics.

**Figure 4:**
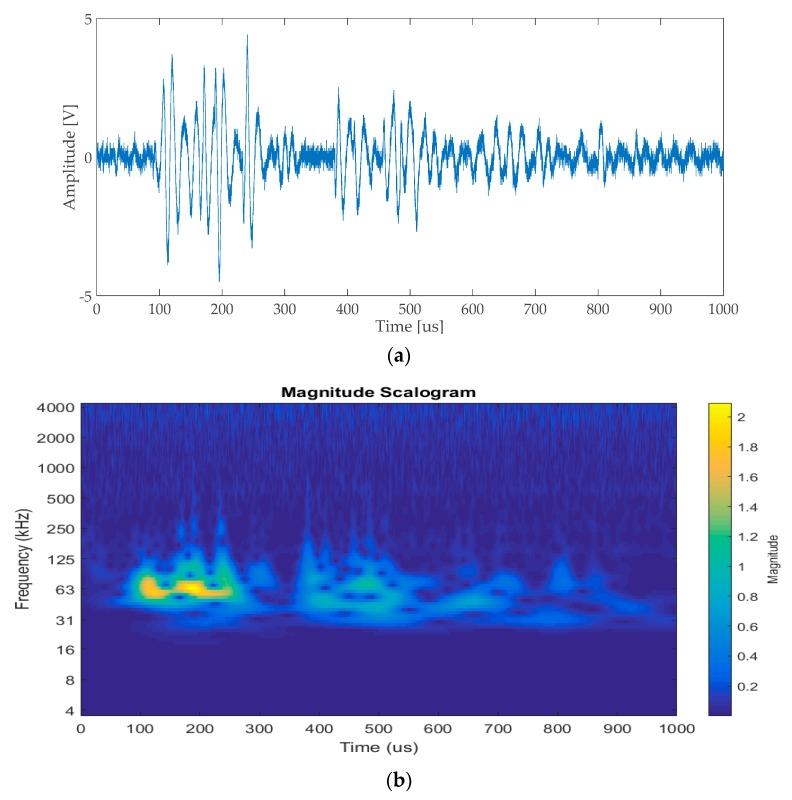
(**a**) Signal generated by a metal sphere impacting on the plate, acquired with an acoustic emission (AE) transducer (Brüel & Kjær Type 8313); (**b**) spectrogram obtained through continuous wavelet transform (CWT).

The experimental setup described in Section 2 was initially used to analyze the impact response signals of the test plate, without using all the sensors that were later exploited to perform impact localization. A single contact transducer was used for this task: a resonant (~200 kHz) Brüel & Kjær Type 8313 acoustic emission (AE). The impact response signal of a low-energy impact (9.17 mJ) was acquired with the scope, and is shown in Figure 4a.

Other measurements performed with two receiving type C sensors (placed at the same position on opposite sides of the CFRP plate) and they confirmed that the impact response signals were mostly constituted by A_0_ mode Lamb-wave packets, while S_0_ signals were remarkably weaker.

### 3.1. Spectral Content of Impact Response Signals

Visual inspection of the time-domain trace of Figure 4a highlighted the presence of a series of wave packets, which could be interpreted as multiple reflections of the impact response signal from the plate edges.

Figure 4b shows the time-frequency plot obtained through continuous wavelet transform (CWT) of the acquired signal. From this spectrogram, it was clear that most of the energy of the signal was mostly confined around 60 kHz–70 kHz, thus suggesting the bandwidth over which the measurement of guided-wave group velocities was to be performed.

## 4. Characterization of Guided-Wave Group Velocities on the CFRP Plate

Guided-wave group velocities along the main directions of the CFRP plate were measured using the method reported in [10,14].

Two type C transducers (Acellent SML-SP-1/4-0) were coupled to the plate surface using an ultrasonic shear-wave couplant (Panametrics NPD-053-8002). One transducer was fixed at the center of the plate and used to transmit Morlet wavelets with different central frequencies, exciting A_0_ Lamb waves that were picked up by the other transducer. The selected center frequency range used for these measurements (20 kHz to 100 kHz) was chosen to cover the results of the spectral analysis performed in Section 3.1.

**Figure 5:**
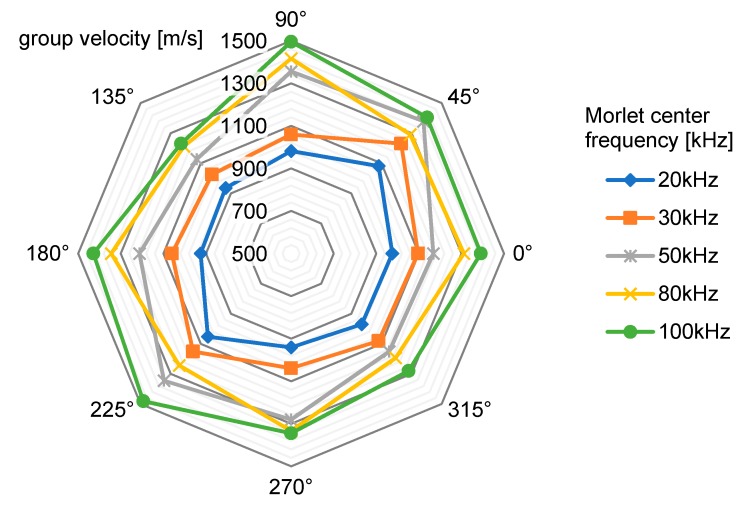
Group velocity measurements for anti-symmetric mode A_0_ on the test plate.

Measurements were performed at all of the selected central frequencies for different directions (following for the reference system of Figure 1), and the results are shown in Figure 5. The plot shows anisotropic characteristics and group velocities in line with the results published in the literature [10].

## 5. An Improved Impact Localization Algorithm

### 5.1. DToA Time-Domain Extraction Techinque Based on Oscillation Selection

**Figure 6:**
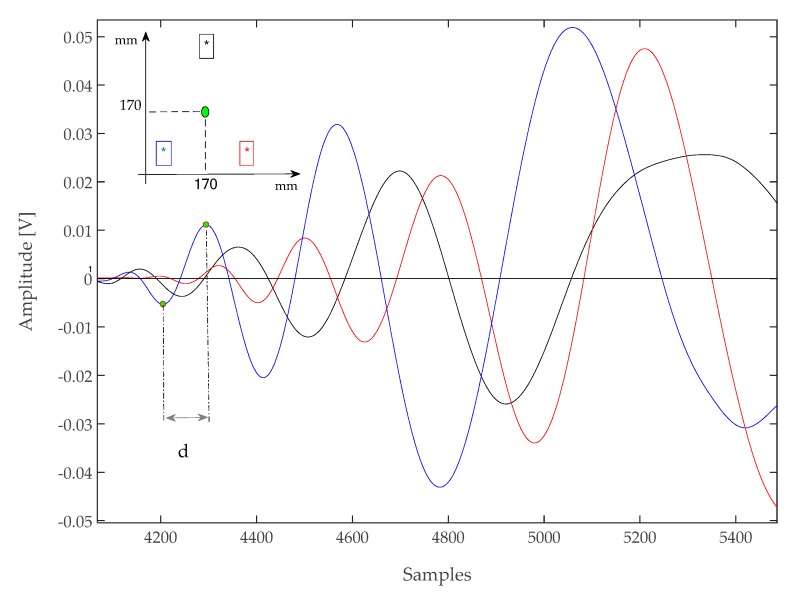
Signal acquired with type A sensors after an impact at (170, 170) mm. Sampling rate 10 MSps. The interval between a previous minimum and the successive maximum of a zero-crossing is marked with “*d*” in the plot.

**Figure 7:**
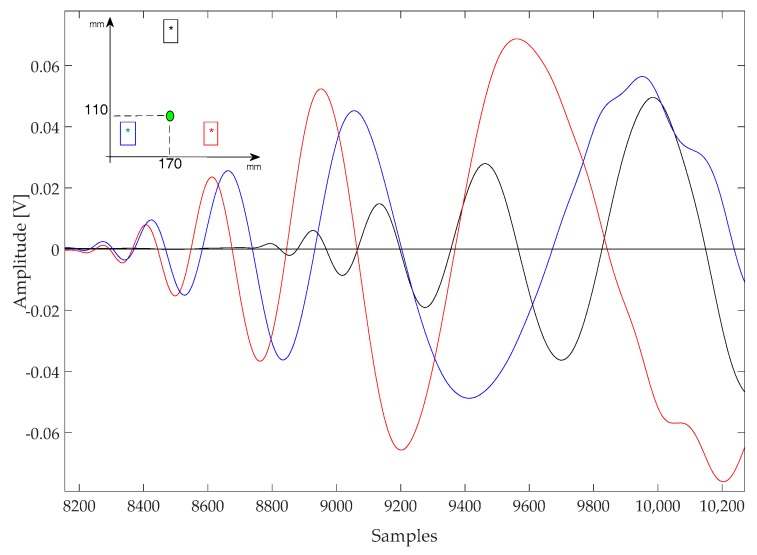
Signal acquired with type A sensors during an impact at (170, 110) mm. Sampling rate 10 MSps.

In general, a low-energy impact generates a complex impulsive waveform with a distinct envelope that can be observed in many signals published in the literature [15,16]. Figure 6 and Figure 7 report the onset of a few impact response signal, all are characterized by a series of oscillatory cycles of growing amplitude. After this initial portion, the shape of signals becomes irregular due to multipath propagation (e.g., reflections from plate edges), mode superposition, saturation of the acquisition electronics, and other phenomena.

An analysis of Figure 6 and Figure 7 shows that each signal has at least three oscillations before being disrupted, and also the different attenuation along different directions due to both the path length and the CFRP laminate stack-up [15]. Those oscillations are easily identified by looking for their zero-crossings. However, given the non-stationary nature of the signals, additional steps had to be taken to ensure a correct identification: simply looking for the zero crossing would also capture sections of the signal before the onset of the oscillatory response, where only noise (and possibly interference) is present. This problem was avoided by setting a threshold higher than the noise level to identify the zero-crossings.

The procedure for setting such a threshold was done automatically on the acquired signals by measuring the maximum noise amplitude from the initial 50 μs of the recorded traces (which are free from any impact-related signal) and using a level four times greater than that value. The actual crossings of the impact response signals could thus be identified reliably without presetting a threshold.

The proposed algorithm thus proceeded by identifying the first threshold crossing, and then searching for the previous local minimum and the successive local maximum of the waveform, and extrapolating their time difference (indicated as “*d*” in Figure 6). This interval depended on the instantaneous frequency of the signal, and could thus be converted in an approximate frequency by taking the inverse of 2*d*. The resulting value, which was dubbed “frequency hook”, was used to characterize the signal swing across the zero-crossings of the waveform. Note that this “frequency hook” parameter does not measure the spectral content of a specific portion of the waveform, because given the non-stationary nature of the waveforms (which are not pure sine waves), their actual bandwidth will be broader.

After the “frequency hook” had been extracted, the corresponding zero-crossing was discarded if its value fell outside the f_min_ = 30 kHz to f_max_ = 80 kHz range. This range was defined empirically, and loosely corresponded to the spectrum shown in Figure 4b. If a zero-crossing was discarded, the process would be repeated on the next one, until a section of the signal that satisfies the “frequency hook” criterion was found, otherwise the algorithm stopped the search and marked the last processed zero-crossing as the time reference for calculating the DToA between the traces.

Regarding the definition of the range ∆f_hook_ = f_max_ − f_min_ for the “frequency hooking”, we observe that this task is done empirically, but it can be replicated for the investigation of different composite materials and structures by using the following general rules:

- The adopted sensors must have a bandwidth including ∆f_hook_
- The impact energy spectrum is high within ∆f_hook_.

### 5.2. Triangulation Formula

After obtaining the DToAs byusing the method described above, the impact position coordinates were found by solving a triangulation scheme with a brute-force minimization approach. The target surface was divided in a quadrangular grid with fixed step size of 1 mm, and an error function *E*(*x_p_*, *y_p_*) was calculated for every one of the grid nodes (*x_p_*, *y_p_*) according to Equation (2), proposed by Kundu et al. in [1]. The coordinates of the sensors are indicated with (*x_i_*, *y_i_*) and (*x_j_*, *y_j_*), while the term DToA*_ij_* represents the quantity (*t_i_* − *t_j_*), that is the difference of the absolute time of arrival detected with the proposed algorithm on the waveforms acquired with sensors *i* and *j*. The absolute minimum of *E*() was then found, and its coordinates represented the best estimate of the impact location.

The minimum number of sensors needed to univocally solve the problem is *N_T_* = 3, corresponding to the experimental conditions described in this paper, but this method can accommodate an arbitrary number of inputs.

(2) $$E\left(x_{p},y_{p\;}\right)=\overset{N_{T}-1}{\underset{i=1}{\sum }}\overset{N_{T}}{\underset{j=i+1}{\sum }}\left|DToA_{ij}\cdot \bar{v\left(\theta ,f\right)}-\left(\sqrt{{\left(x_{i}-x_{p}\right)}^{2}+{\left(y_{i}-y_{p}\right)}^{2}}-\sqrt{{\left(x_{j}-x_{p}\right)}^{2}+{\left(y_{j}-y_{p}\right)}^{2}}\right)\right|$$

The DToA values extrapolated from the time-domain traces needed to be converted into a distance to solve Equation (2), requiring the knowledge of a propagation velocity *v*. However, as explained above, this parameter depended on both the frequency (since guided waves are dispersive) and direction (the guiding medium is anisotropic), becoming a function v(θ,f). A workaround to avoid this complication was found by replacing v(θ,f) with a value averaged over both of the arguments from the data reported in Figure 5, calculated by considering only the group velocity profiles within the bandwidth of interest. The resulting average velocity value was $\bar{v\left(\theta ,f\right)}$ = 1223 m/s, and was used in (2) to calculate all the localization results presented throughout this paper.

## 6. The Performance of the Proposed Algorithm

To evaluate the performance of the proposed algorithm, five impact tests were carried out at different coordinates on the CFRP test plate using the setup described in Section 2. The five positions numbered #1 to #5 (shown in Figure 1) had coordinates (*x*, *y*): (170, 170) mm, (170, 230) mm, (170, 110) mm, (110, 170) mm, and (230, 170) mm, respectively. Every impact test was repeated ten times for each combination of impact point and sensor type.

### 6.1. Localization Improvement with the Proposed DToA Extraction Method

The beneficial effects of the proposed algorithm are best understood by analyzing how certain impact response signals were processed, and how the DToAs were extracted.

**Figure 8:**
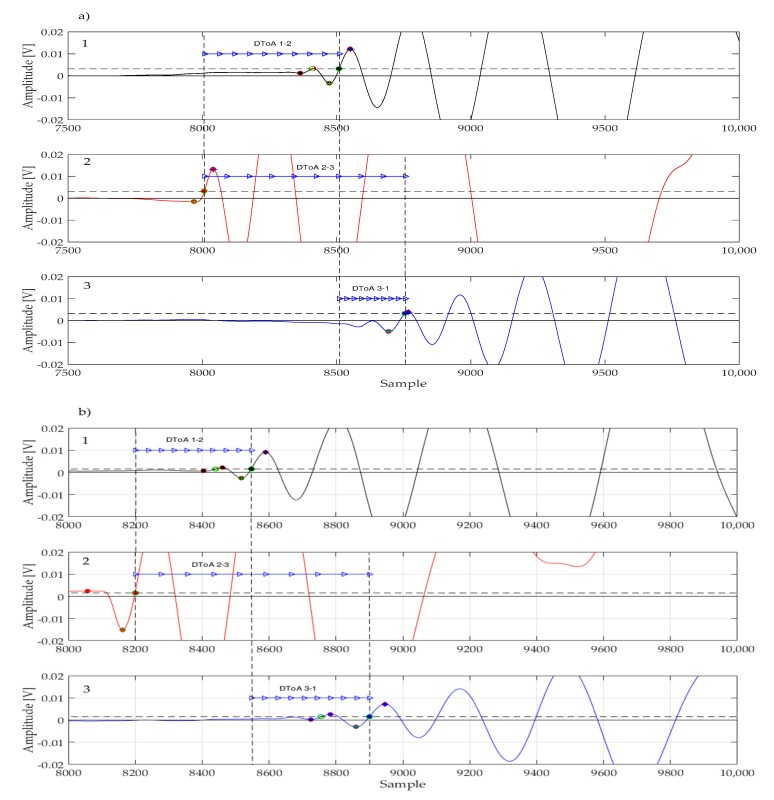
Signals acquired with type A sensors after two impact events: (**a**) impact at (110, 170) mm; (**b**) impact at (230, 170) mm. The sampling rate was 10 MSps.

**Figure 9:**
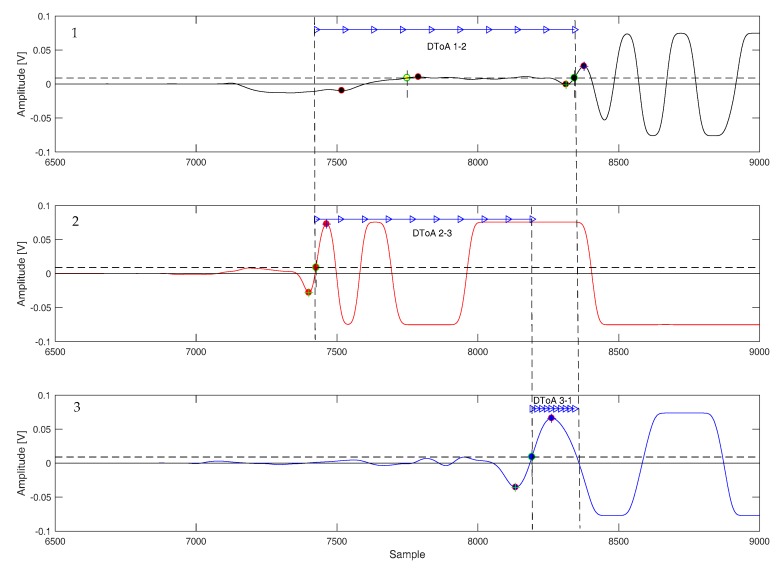
Signals acquired with type B sensors after impact at (170, 110) mm. Sampling rate 10 MSps.

Figure 8a,b shows the waveforms acquired during impact tests #4 and #5 with type A sensors. We can observe that the signals present some sort of low-frequency oscillation just before the onset of the impact response transient. This spurious signal may present itself with different shapes and amplitudes (see for instance Figure 9) and it is detrimental when using a simple thresholding technique, as it could be easily mistaken for the leading edge of the impact response. Using the method proposed in Section 5.1, spurious threshold crossings are ignored and the DToA is extrapolated from the correct portion of the acquired signal.

### 6.2. Summary of Experimental Results

Table 1 summarizes the results of the impact experiments performed on the CFRP plate obtained by performing the proposed algorithm on the acquired data. It should be noted that the repeatability of the impact test rig, mentioned in Section 2, contributes to the localization error.

Moreover we can evaluate the improvement of the proposed algorithm compared to the case of the DToA estimation with the automatic threshold. 

## 7. Discussion

An analysis of the complex nature of the impact response signals acquired with various sensors highlighted the need for robust processing in the estimation of DToA. The authors thus developed an algorithm capable of extracting an accurate DToA by exploiting the oscillatory characteristics of the received signals.

To solve the impact triangulation Formula (2), two parameters that depend on the characteristics of both the medium and of the guided waves were needed: the DToAs and the propagation velocity. Measurements showed that the dispersion and anisotropy caused a variation of up to ±22% of the velocity profiles within the bandwidth of interest and over different directions. The extraction of DToA was also affected by error, however acting on it does not require extensive characterization of the guiding medium, and therefore can represent an overarching improvement of impact triangulation accuracy.

Table 1 shows that the localization results can be improved by applying the proposed DToA extraction algorithm, instead of the automatic threshold-based technique.

To confirm the results shown in Table 1, further experiments were performed with a heavier impactor sphere to increase the impact energy from 35 mJ to 600 mJ; metal objects with different shapes (like bolts and nuts) but comparable dimensions and mass were also tested. Even in those cases, the algorithm could find frequency hooks within the range of 30 kHz–80 kHz and extrapolate a DToA, obtaining localization accuracies comparable to those reported in Table 1.

## 8. Conclusions

This paper presented a novel method for recognizing the differential time-of-arrival of impact response signals that improves the impact localization accuracy when using triangulation. This method was applied to guided-wave impact localization in CFRP plates, where dispersive and anisotropic behavior are non-negligible.

It was found that approximating the propagation velocity of the received signals with a constant value has an influence on the results similar to the error introduced by a DToA estimated affected by a significant error. Missing the correct zero-crossing can lead to large errors, especially in the presence of spurious contributions within the impact response signals. Therefore, improving the DToA extraction method resulted in a net improvement of the localization performance, comparable to knowing the actual propagation velocities to a good accuracy.

The proposed method was successfully applied to different type of piezoelectric sensors without tuning its parameters to the specific sensors type.

Finally, the proposed method for the DToA estimation can be integrated with other methods already presented in the literature that provide an accurate estimation of the v(θ,f) to improve the overall localization accuracy of the impact.

## Figures and Tables

**Table 1:** Comparison of the impact localization results obtained on the same data using a simple threshold method vs. the proposed algorithm.

Sensor Type	Number of Tests	DToA Extraction Method	Average Error	Standard Deviation
			(mm)	(mm)
Type A (Circular PVDF)	32	Threshold	20.44	13.78
		Proposed	20.28	8.92
Type B (P-876.SP1)	24	Threshold	31.33	17.511
		Proposed	23.17	10.41
Type C (SML-SP-1/4-0)	22	Threshold	19.32	10.74
		Proposed	17.05	8.81

## References

[1] Kundu T. (2014). Acoustic source localization. Ultrasonics.

[2] Jiao J., He C., Wu B., Fei R., Wang X. (2004). Application of wavelet transform on modal acoustic emission source location in thin plates with one sensor. Int. J. Press. Vessels Pip..

[3] Ciampa F., Meo M. (2010). A new algorithm for acoustic emission localization and flexural group velocity determination in anisotropic structures. Compos. Part Appl. Sci. Manuf..

[4] Ciampa F., Meo M., Barbieri E. (2012). Impact localization in composite structures of arbitrary cross section. Struct. Health Monit..

[5] Garofalo A., Testoni N., Marzani A., Marchi L.D. Wavelet-based Lamb waves direction of arrival estimation in passive monitoring techniques. Proceedings of the IEEE International Ultrasonics Symposium (IUS).

[6] Nakatani H., Kundu T., Takeda N. (2014). Improving accuracy of acoustic source localization in anisotropic plates. Ultrasonics.

[7] Mueller I., Larrosa C., Roy S., Mittal A., Lonkar K., Chang F.-K. An integrated health management and prognostic technology for composite airframe structures. Proceedings of the Annual Conference on Prognostics and Health Management.

[8] Bulletti A., Giannelli P., Calzolai M., Capineri L. (2016). An Integrated Acousto/Ultrasonic Structural Health Monitoring System for Composite Pressure Vessels. IEEE Trans. Ultrason. Ferroelectr. Freq. Control.

[9] Bellan F., Bulletti A., Capineri L., Masotti L., Yaralioglu G.G., Degertekin F.L., Khuri-Yakub B.T., Guasti F., Rosi E. (2005). A new design and manufacturing process for embedded Lamb waves interdigital transducers based on piezopolymer film. Sens. Actuators A Phys..

[10] Galeazzi R. Studio Sperimentale Finalizzato al Monitoraggio di Difettosità interne in Laminati CFRP per Utilizzo Aeronautico, Mediante Approccio Ultrasonoro con Onde di Lamb. https://www.politesi.polimi.it/handle/10589/83381.

[11] Capineri L., Bulletti A., Calzolai M., Giannelli P., Francesconi D. (2014). Arrays of conformable ultrasonic Lamb wave transducers for structural health monitoring with real-time electronics. Procedia Eng..

[12] Capineri L., Bulletti A., Calzolai M., Francesconi D. (2014). A real-time electronic system for automated impact detection on aircraft structures using piezoelectric transducers. Procedia Eng..

[13] Giannelli P., Bulletti A., Capineri L. (2017). Multifunctional Piezopolymer Film Transducer for Structural Health Monitoring Applications. IEEE Sens. J..

[14] Wang L., Yuan F.G. (2007). Group velocity and characteristic wave curves of Lamb waves in composites: Modeling and experiments. Compos. Sci. Technol..

[15] Adams D. (2007). Health Monitoring of Structural Materials and Components: Methods with Applications.

[16] Prosser W.H., Seale M.D., Smith B.T. (1999). Time-frequency analysis of the dispersion of Lamb modes. J. Acoust. Soc. Am..

